# Unlocking the potential for engaging men to improve reproductive, maternal, and neonatal health in Karnali Province, Nepal

**DOI:** 10.1186/s12889-022-14534-6

**Published:** 2022-11-16

**Authors:** Khem Narayan Pokhrel, Dipendra Singh Thakuri, Nana Apenem Dagadu, Roma Balami, Matrika Sharma, Rajan Bhandari

**Affiliations:** 1 Partnership for Social Development Nepal, Maharajgunj, Kathmandu, Nepal; 2 Development and Research Service International Nepal, Khumaltar-15, Lalitpur, Nepal; 3 Save the Children International (SCI), Airport Gate, Sinamangal, Kathmandu, Nepal; 4 Save the Children US, Washington, DC, USA

**Keywords:** Healthy transitions, Adolescent girls and young women, Male engagement, Reproductive, maternal and neonatal health, Nepal

## Abstract

**Background:**

Adolescent girls and young women (AGYW) often experience early childbearing and have poor utilization of reproductive, maternal, and neonatal health (RMNH) services in Nepal. Involving men in such services has been increasingly recognized globally to improve gender-equitable reproductive health behaviour in husbands. This qualitative study assessed the implementation of Healthy Transitions’ male engagement interventions in Karnali Province, Nepal which were implemented to improve gender-equitable attitudes, and supportive RMNH care-seeking behaviors among the husbands of young women.

**Methods:**

We conducted a summative qualitative study that included in-depth interviews with 12 AGYW as primary beneficiaries and their husbands (*N* = 12) and in-laws (*N* = 8). In addition, key informant interviews were conducted with health workers (*N* = 8), local government representatives (*N* = 4), members of Health Facility Operation and Management Committee (*N* = 8) and project implementers (*N* = 12). Due to COVID-19-related travel restrictions and lockdowns, all interviews were conducted via phone calls and online consultation. Data were analyzed using multistage coding and thematic content analysis.

**Results:**

AGYW, their husbands, in-laws and health workers were receptive to the Healthy transitions’ male engagement initiatives. They perceived that the project contributed a momentum to facilitate men’s gender-responsive behaviour. Many participants reported that male engagement interventions, including home visits, community dialogues, and social events improved husbands’ support for their wives during menstruation, pregnancy, and childbirth. The activities also facilitated spousal communication and improved the couple’s decision-making for family planning use. Women reported that improved support from their husbands increased their self-confidence.

**Conclusions:**

This study sheds light on the role of male engagement strategies to improve RMNH in a context where inequitable gender norms and roles are highly prevalent. Our findings highlight the potential to improve RMNH by addressing barriers to male engagement.

## Background

Adolescent girls and young women (AGYW) often experience poor access to reproductive, maternal, and newborn health (RMNH) services in Nepal. Various factors such as gender inequality, societal norms, and lack of support from their husbands and families [1] contribute to poor health service utilization. Transition to early marriage and childbearing among AGYW put them at risk of their morbidity and the health and development of their children [2]. Despite the legal age of marriage in Nepal being 20 for both sexes, the average age for marriage for girls is 17.9 years and 27% of the adolescents aged 15–19 years are already married [3]. In addition, 17% of the adolescent girls aged 15–19 years were either pregnant or already had their first child, and modern contraceptive use is low (15%) among married adolescents. Adolescents also have the highest unmet need for family planning (35%) compared to unmet need (24%) among married women of reproductive age. RMNH status of Karnali Province, in the hilly region of the mid-western part of Nepal, remains poorer compared to the national level. The province has the youngest median age at marriage (17.4 years) and at first birth (19.8 years) and almost 15% of the adolescents aged 15–19 years had already begun childbearing [3]. Also, Nepal’s Demographic and Health Survey 2016 shows that the Karnali province has the second-highest total fertility rate (2.8) and adolescent childbearing rate (19%) and a lower-skilled birth attendance rate (36%) compared with the national average total fertility rate (2.3), adolescent child bearing rate (17%) and, skilled birth attendance rate (58%). Early marriage, early childbearing, gender inequality, existing harmful social norms, and practices may be the underlying cause that resulted in poor utilization of RMNH services.

The positive male engagement has been shown to increase a woman’s confidence in attending health-care services on her own [4], responding to delays in seeking care [5], increasing family planning and other health care discussions and appointments [6]. Past evidence showed that if both partners are equally engaged in healthcare decisions and family planning uptake, they will be more likely to create a home environment that will eventually improve their health and the health of entire family [6]. Evidence is available that male engagement strategies were effective to improve the utilization of RMNH services in various settings and thereby positively influence the maternal and child health outcomes [7–9].

The government of Nepal has recognized the role of men in family planning and maternal health in its policy documents. For instance, men are receiving family planning services such as condoms and vasectomy free of cost from public health facilities [10]. Also, Nepal’s Safe Motherhood and Reproductive Health Rights Act 2018 is providing male service holders with 15 days of paid paternity leave, which is intended to provide a caregiving role to men [11]. Nepal Safe Motherhood and Newborn Roadmap 2030 [12], which has also identified that men’s decision-making and gender-related behavior affects the access and utilization of maternal and newborn health services. However, Nepal’s existing policies and strategies do not explicitly call for men’s or husbands’ role in RMNH services despite it is being crucial for improving access to and utilization of such services. In addition, limited studies identified the extent and factors related to male involvement in reproductive and maternal health in Nepal [13–15] and only a few programs address the unique health needs of young married adolescents in Nepal. However, there remains a need to understand the role of male engagement strategies in RMNH in the Nepalese context and the perspectives of key health workers and community stakeholders on the implementation process of male engagement interventions. This study aimed to assess the perception of beneficiaries (AGYW and their husbands and in-laws) and key stakeholders such as government officials and health workers about the male engagement interventions of Healthy Transitions for Nepali Youth Project (Healthy Transitions) and its impact on male engagement in RMNH in the resource-poor setting of Karnali province.

### Healthy transitions’ male engagement interventions

**Fig. 1:**
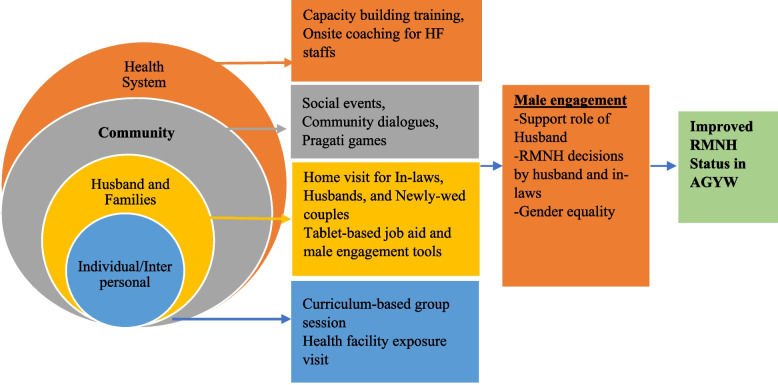
Intervention design of Healthy Transition Project

To address the transition of AGYW on marriage and childbearing, Save the Children International Nepal designed and implemented healthy transitions male engagement interventions. Four local partner non-governmental organisations (PNGOs) implemented the interventions in collaboration with government counterparts and related stakeholders from 2018 to 2021. The project drew on lessons learned from previous evidence-based interventions implemented by Save the Children in Nepal, including My First Baby [16] and *Pragati* games, the locally designed fertility awareness games from Fertility Awareness for Community Transformation Project [17]. The program targeted ten geographical clusters in each district, covering 40 health facilities and their catchment area. Aligned with the socio-ecological model, Healthy Transitions introduced three gender-transformative strategies at interpersonal, family and community level: interactive games in small groups, home visits through tablet-based videos, and community dialogues/events (Fig. 1). The community level activities aimed to improve husbands’ gender-equitable attitudes and supportive RMNH-seeking behaviours, and contribute to the goal of improving RMNH among AGYW. The intervention at the individual and family level involved home visits by the female social mobilizers, who completed auxiliary nurse midwives or staff nurse courses. These mobilizers were trained by project staff on male engagement interventions. Each social mobilizer was intended to visit 20 homes of married AGYW per month to engage male partners, young couples, and in-laws in the dialogue using a tablet-based job aid, approved by the National Health Education Information and Communication Centre (NHEICC), Department of Health Services, Ministry of Health and Population. The job aid was comprised of six short videos that addressed: 1) gender equality in the household; 2) spousal engagement in newborn and maternal care; 3) mother-in-law support for pregnant women during a husband’s absence due to migration; 4) couple FP conversations after childbirth; 5) participation of women in household decision making; 6) support for girls’ education. While visits were intended to be conducted monthly to each enrolled participant’s household using this tablet-based job aid, there was a delay in finalizing the videos. In the interim, social mobilizers conducted the visits using male engagement tools adopted from the USAID funded Fertility Awareness for Community Transformation project’s Roving Auxiliary Nurse Midwife Service Delivery Intervention [18] in the months before the videos were ready. Community-level interventions involved social events and community dialogues. Social mobilizers organized various events such as sports, celebrations, quiz or game shows, and theatrical performances biannually to share the Healthy Transitions’ contents more widely and expose communities to the topics covered therein. The intent was to increase knowledge, generate discussion, and spur communities to work through understanding of attitudes and beliefs to support more gender-equitable norms, such as increased participation in household decision making (including related to finances and FP), and to provide support to AGYW in seeking information and services related to RMNH. These social events were organized with migration patterns so that activities were held when men were likely to be home.

Following pre-implementation training, social mobilizers conducted dialogues with community leaders and other relevant stakeholders in each district using the NHEICC-approved community dialogue and reflection tool and *Pragati* games. These dialogues engaged the community members four times per month with the reflection and action to reduce child marriage, improve RMNH outcomes, and support adolescents and youth to live in more supportive and gender-equitable communities.

## Methods

### Study design and setting

To explore the views of the community on the Healthy Transitions’ male engagement  interventions, the study team conducted a qualitative study between June and August 2021 after 15 months of the project implementation. We used semi-structured tools to capture directly their experience leading the project in their community including achievements, program influences and barriers in terms of male engagement initiatives. The study was conducted in Nine Healthy Transitions intervention Rural/Municipalities of the Dailekh, Kalikot, Jajarkot, and Surkhet districts of Karnali province.

### Recruitment and sampling

For this study, we recruited the study participants from each district of project implementation that reflected the diversity of the participants from Healthy Transitions catchment area and ensured data saturation. We purposively sampled a total of 64 individuals including AGYW, husbands and in-laws of AGYW, health workers, representatives of local government and health facility operation and management committee, and project staffs from four Healthy Transitions project districts. In each district, we conducted 8 In-depth interviews (IDIs) with the direct beneficiaries such as AGYWs and their husbands and in-laws and 8 Key Informant Interviews (KIIs) with community stakeholders such as health workers, representatives of local governments and project implementers. The sample size was determined to capture the rich information from beneficiaries and various stakeholders and 16 participants were selected equally distributed among the four project implementation districts of Healthy Transitions project (Table 1).

**Table 1:** Distribution of IDI and KII participants by districts

Participants	Districts				Total Sample	Method of data collection
Beneficiaries and stakeholders	Kalikot	Jajarkot	Surkhet	Dailekh		
Adolescents and young women	3	3	3	3	12	IDI through Phone
Husbands	3	3	3	3	12	IDI through Phone
In-laws	2	2	2	2	8	IDI through Phone
Health workers	1	1	1	1	4	KII through Phone
Health coordinators	1	1	1	1	4	KII through MS Teams
Ward Chair/HFOMC representative (4-chair and 4 youth)	2	2	2	2	8	KII through phone
R/Municipalities Chair	1	1	1	1	4	KII through phone
Project staffs						
Project coordinators	1	1	1	1	4	KII through MS Teams
Social Mobilizers	1	1	1	1	4	KII through Phone
Mentors	1	1	1	1	4	KII through Phone
**Total**	**16**	**16**	**16**	**16**	**64**	KII and IDI

### Data collection

Interview guidelines for KII and IDI tools were developed for diverse types of participants and covered the acceptability, perceptions, barriers and reflections about Healthy Transitions' male engagement interventions. We also reviewed the project documents and reports, monitoring data, and social behavioural change communication (SBCC) materials of the project to prepare the interview tools. The interview tools were pretested in similar settings through phone and some modifications were made before initiating the interviews with actual participants of the study. Four Researchers conducted KIIs and IDIs through phone contact and online meetings. The researchers received 3 days of training on tools, ethical procedures, and interview skills. Around 30-40 minutes interviews were conducted in Nepali language and recorded with the permission of the participants.

### Data analysis and narrative synthesis

The interviews were recorded after receiving verbal consent from the participants. English translation of Nepali audio transcripts was done by the research team on the same day of the interview. Deductive coding was used to develop a pre-established codebook, which was supplemented as themes emerged following data collection. The subset of interviews was double coded, differences in coding were discussed amongst the study team members and the coding schema was revised accordingly, and subsequently applied to the subset. Differences were resolved by consensus, and the schema was applied to the remaining interviews. The thematic framework containing central themes and subthemes was prepared. The verbatim and quotations were used to demonstrate the perception of the respondents. The transcription files were uploaded in Taguette [19], to identify quotes representing different aspects of the themes, and the number of participants mentioning them. Key findings from the interviews were summarized by the study objectives and illustrated with the use of participant’s quotes.

## Results

### I. Socio-demographic characteristics of primary beneficiaries involved in interview

We have collected the demographic information of primary beneficiaries of the intervention only. The mean age of primary beneficiaries (*n* = 12) was 22.2 (SD = 2.8) years and mean age at marriage was 18.3 (SD = 1.6) years. About 41.7% were from the Dalit community followed by 33.4% of Brahmin/Chhetri, and 16.7% were Janajatis. Regarding the number of children, 41.7% had one child and 33.3% had two children, and 17.7% had two children. One woman had no child. In our sample, women (83.3%) and their husbands (83.3%) had education level at Grade 11 and above. Most of the women (75%) were either housewives or and engaged in agriculture. Husbands of the interviewed women were engaged in agriculture (25%), manual labor (16.7%), and in the service sector (33.3%). One respondent reported that her husband is unemployed and another husband of next women was a student.

### II. Perception of beneficiaries and stakeholders on male engagement interventions

#### Perception of AGYW

Most of the married women perceived that the videos shown during the home visits were more contextual and based on the real stories from their community, which supported and strengthened gender-equitable relationships between husbands and wives as well as among mother in-laws. Some women further highlighted that men’s behaviour towards pregnant women had changed because home visits made the husband and other family members more receptive to the program and were inquisitive about the various topics of RMNH.

“The videos shown by sisters were developed based on the stories of our community. My husband and I have seen three videos and liked them all. All videos were helpful for me; my husband, and in-laws understood about our roles and responsibilities during pregnancy and after delivery.” (AGYW, IDI)

#### Perception of husbands

Majority husbands appreciated that the exposure to the Healthy Transition activities was beneficial and completely new for them. They highlighted that the home visits provided them an opportunity to internalize their roles and responsibilities for their wives’ and children’s health.

“The videos were good and had touched my heart, and were based on the real stories of our community. I have never seen such activities in our community before. Now, I am conscious about my role and responsibilities for my pregnant wife and my small children.” (Husband, IDI)

Most of them also perceived that their knowledge regarding family planning, menstrual hygiene, nutrition education, care during pregnancy, and service utilization from health facilities have been increased. They shared that they not only learned the importance of RMNH behavior during the home visits and community dialogues but also learned from their wives. They also themselves changed their mindset on how they could be helpful to a woman to improve her menstrual hygiene, ANC check-up, and use of family planning. Men responded that they were also equally involved in taking care of their children.

“I knew how to speak with everyone with confidence. I knew how to maintain birth spacing. The events of the project were so helpful not only for knowing something new but also being able to teach other people as well.” (Husband, IDI)

#### Perception of stakeholders

Most of the health workers and ward chairpersons were familiar with Healthy Transition male engagement strategies. The ward chairpersons reported that the home visits through videos and community dialogues were appropriate in their community specially to teach husbands and in-laws about RMNH issues and develop gender equitable behaviours among them. They further added that these are practical and effective tools to influence them and other community people especially to improve the health seeking behaviour of husbands and mother in-laws from marginalized groups. The ward chairpersons have suggested continuing these activities through local health facilities even after the completion of the Healthy Transitions project.

“I had a chance to participate Pragati games facilitated by the sister of Health Transition project in our community. In my opinion, these types of activities are practical and based on the life experience of our community people … .and I believe that these will be continued through sisters of health post or female community health volunteers of our community. The women do not go to the health post for check-up after child birth and family members do not allow them to visit health posts, … … in this context the home visit by social mobilizers is very helpful and effective to save the lives of the children and mother.” (Ward Chair, KII)

Similarly, health workers believed that engaging family (husband and in-laws) members and community influential people through the home visits and community dialogues bridged the gap between the service providers and beneficiaries and also created positive momentum for engaging husbands in RMNH.

“The community dialogue was unique to me. I think the home visits by social mobilizers connected husbands with health facilities that facilitated husbands for their supportive behaviour towards their wives and children. Healthy Transition project has proved to be beneficial not only to the adolescent girl but also to all the community members.” (HW, KII)

### III. Perceived changes brought by healthy transition male engagement interventions

#### Improved healthy and gender-equitable RMNH behaviour among AGYW, their husband, and families

The women reported that they improved their behaviours on birth preparedness, nutrition of women during pregnancy and post-partum check up, nutrition requirements of infants and children including the practice of breastfeeding after being exposed to the interventions. They also learned about the fertile period and safe days during menstruation so that they could prevent themselves from unwanted pregnancy. The women reported that their husbands and in-laws were also sensitized about the benefits of delaying marriage, ANC check-up and delivery at the health facility. They have further added that their husbands and in-laws had increased their support during pregnancy.

“My husband and mother-in-law encouraged me to go for my ANC check-up when I was pregnant and they supported me to go for delivery at the health post rather than delivering at home.” (AGYW, IDI)

#### Increased RMNH discussion among couples

Most of the women expressed that the project activities especially home visits and community dialogues created anenabling environment for them to share and discuss any RMNH issues with their husbands, in-laws, and health workers.

“I was very hesitant to speak in front of strangers before. But after having home visit of sisters, I am now more confident and can put up my views in front of other people. I can share any problems or information with my husband and family members. I can share what I learned with other peoples of the community as well”. (AGYW, IDI)

Men also changed their practices such as they initiated the discussion about the fertility and family planning methods among themselves, with their spouse and family members.

“We used to spend a little time to discuss our fertility and family planning-related matters. After participation in Healthy Transition activities, we started discussing more fertile windows, safe days to prevent pregnancy, and benefits of family planning methods.” (Husband, IDI)

Similarly, most of the AGYW highlighted that their husband changed their perception regarding sex determination of children after playing the Pragati game during community dialogue events.

“My husband used to say that the sex of the child is determined by the women and we were always blamed in the community if we give birth a girl child. He now believes that it would be a great injustice to blame women for bearing a girl child.” (AGYW, IDI)

One AGYW shared that she increased her frequency of visits to health facilities. Some health workers thought that the couple increased discussion between themselves to decide whether giving birth or choosing the appropriate method of contraceptives and their benefits.

“The couples have now understood that they should prepare themselves and discuss with health workers before giving birth to a child. They started discussing each other before choosing an appropriate method of contraception.” (HW, KII)

#### Improved menstrual hygiene management practices

AGWY expressed that their family members were more open towards menstruating women. They were more conscious of the nutrition, hygiene, and workload of women during pregnancy and menstruation periods. Women felt that their husband and in-laws changed their perceptions and practices about menstruation and harmful cultural practices such as Chhaupadi practice (cultural practice of using makeshift shelter during menstruation in certain geographical region of Nepal),

“Now, my family members give me buffalo milk and curd, and other nutritious foods during my period. They don't force me to go for chhaupadi practice while in the period. My husband is also becoming more supportive during my period.” (AGYW, IDI)

Women started using sanitary pads and are allowed to sleep at home. Those who used cotton clothes also started washing them frequently and drying them in sunlight before use.

“Earlier, my wife used to get shy using pads (during menstruation). She now uses sanitary pads. … We also had a tradition of (women's) seclusion (outside the home) for five days during menstruation, Chhaupadi. We have abolished such practices after realizing that it is not good.” (Husband, IDI)

#### Men’s changing attitude towards the supportive role

The supportive role of men was observed after the implementation of the Healthy Transition project. Most of the women expressed that their husbands started supporting them (wives) in household chores during pregnancy and delivery and primarily accompanying their spouse for ANC and family planning services. They further added that the husband’s attitude also changed towards gender-responsive behaviour such as they started communicating about women’s fertility, family planning, and pregnancy.

“After the project started, my husband cared for me a lot and provided me with nutritious food to eat when I was pregnant. He also goes with me when I go for ANC check-up” (AGYW, IDI)

The health worker reported that there were not any specific activities to engage males in a safe motherhood program before the project. After the Healthy Transitions, where husbands of the pregnant women were involved in the social events and community dialogues conducted in the health post, men were accompanying their wives during ANC visits. They further added that men also realized that they needed to have an equal role in maternal and child healthcare services.

“Earlier, men didn't accompany their wives for check-ups or even during delivery but now, husbands come for their wives’ regular ANC check-up. Likewise, now, we have witnessed many fathers coming to vaccinate their children while earlier, only females would come for such purpose.” (HW, KII)

### IV. Barriers and enablers in engaging males during project implementation

#### Barriers

##### Migration of men and their engagement in out of household work:

Reaching migrant men and those who used to work outside of their homes was difficult despite those men being interested to participate in-home visits, community dialogues, and social events. The majority of adolescent and young men used to migrate to India and middle-east countries for their employment. Similarly, husbands’ involvement in daytime work as daily wage labourers were outside of their homes. Some male members even did not know whether there were home visits although their family members had seen some videos related to RMNH.

“It was difficult to meet the male participants during the home visits who had gone to India and other countries to earn for their living.” (SM, KII)

##### Limited activities targeting men:

Some males and project staff (PC, SM, and mentors) felt that there should be group sessions and other activities for males as that was implemented for women. The male members were invited to community dialogues, social events sessions and participated in the *Pragati* games.

“I was feeling that the program was being less effective because we reached mostly wives. After our efforts, husbands also participated in social events and played the Pragati games, they liked it very much and they shared those learned things with their family members and friends.” (SM, KII)

##### Socio-cultural barriers:

Some of the male members felt shy and were hesitant to participate in any activities of the project. Participants often held back to discuss issues related to women’s health and open up their issues. They also had a fear about the disclosure of their reproductive issues as they thought it was their private matter. In some cases, gender barriers were the major issues to organize the activities because some men were reluctant to discuss much in front of women mentors.

“When a male member changed his attitude and tried to help his wife, his friends and society gossiped and talked negatively about him. People started calling him joitingrey (One who is submissive towards his wife).” (AGYW, IDI)

Likewise, the male members used to hesitate in participating in the social events or home visits. One of the reasons might be that they felt uncomfortable discussing sexual and reproductive health issues with their family members. Other reasons might be that they did not perceive it good to watch videos or discuss sexual and reproductive health among the family members. Social mobilizers experienced such challenges in the beginning, but they overcame them when they explained the process clearly.

“We used to visit the newly married couple at their home . At the time of the video shows, newly married couples were not comfortable watching the video. They used to say, they didn't have time and they would watch videos later. Later, after we visited them repeatedly, they understood.” (SM, KII)

The social fabric of the relationship between brother-in-law and sister-in-law such as not sitting together was prevailing in their society that resulted the poor participation of male members, especially in community dialogues.

“When we participated in the meetings, the sisters were facilitating all the events and we felt uncomfortable asking questions about our problem. However, the topics discussed during community dialogues and social events were interesting and useful for me and my family.” (Husband, IDI)

#### Enablers

##### Interventions appropriate to engage a newly married couple:

The motivation of husbands and positive support from family members enabled AGYW to engage men in RNMH activities. When husbands were supportive, women’s participation was high. Women discussed the knowledge with their husbands that they learned in the group sessions, which was one of the enabling factors for engaging males in the RMNH activities. *“My husband changed his thoughts and he allows me to participate in Pragati games and sometimes he attends the session.” (AGYW, IDI).*

##### Increased perceived need for utilization of RMNH services:

Women were aware of the benefit of RMNH services through their engagement in group sessions. They also learned about the provision of cash incentives that is being provided to mothers who completed four ANC visits and delivered at the institution. Health facility visits were effective where the social mobilizers and mentors used to take the AGYWs to the nearest health facilities, and they used to describe the services provided from health facilities. Such visits acted as a bridge to limit the gaps between the demand and supply-side barriers in access to and utilization of healthcare services.

“The utilization of health facility services has been increased. It is not only because of incentives, but also because of the understanding of the importance of ANC check-up and institutional delivery.” (Local government representative, KII)

## Discussion

This study showed the impact of community based interventions of male engagement in improving RMNH outcomes in the area where gender norms attribute to poor health of adolescent and young women. The findings of this study shed light on major gender and socio-cultural challenges faced by adolescent girls and young women to access RMNH services. Similarly, the male engagement component of Healthy Transitions project found to be effective to respond traditional and cultural beliefs [14, 20, 21] of that prevents Nepalese husbands from being involved in reproductive and maternal health care. This intervention was implemented in the area where such beliefs and practices are highly prevalent [20, 22]. Healthy Transitions interventions significantly contributed to change the perspectives of husbands regarding their supportive roles and responsibilities during pregnancy and after child birth. In addition, husbands have adapted positive behaviors related to menstruation and Chhaupadi practices which was highly prevalent in our study area [20]. Men are usually not allowed to touch adolescents and women during menstruation. Such belief is often harmful and women are being stigmatized that resulted in poor utilization of RMNH services [23]. The project provided the platform for discussion about such cultural barriers among men, and they realized that it was a gender-related bad cultural practice when they participated in community dialogue and social events. The intervention facilitated discussion between couples about contraceptives, menstrual practices and supportive role during pregnancy and delivery as shown in other studies [7–9], which could have positive impact in the field of family planning and utilization of maternal health services, immunization and nutrition [15, 24, 25]. Furthermore, the intervention found to be effective to transform men’s attitudes towards gender-responsive behaviour such as they started communicating about women’s fertility, family planning, and pregnancy which is consistent with the previous studies [7, 26] which have shown that male involvement programs had the potential to generate changes in men’s attitudes and behaviour.

The findings suggested that the interventions especially the home visits through the tablet-based job aid can help husbands become more conscious about contraceptive use, care of wives during pregnanancy and child birth. Continuation of these activities through local health facilities could have been great impact in the utilization of RMNH services in the area where the husband’s role during pregnancy, child birth and post-partum is limited [25].

Similarly, the family discussed together gender equality, the male’s role in supporting the spouse in accessing health services, and related information. Gender equality continuum tools state that men will be more aware of the gender when they are engaged and lead [27].

Despite the successful implementation, some of the implementation barriers exist in the project as men’s involvement in maternal and child health related interventions is a complex that is linked to the socio-cultural factors and dynamics of the society [14]. It was difficult to reach the migrating men in India and those who were working out of household work because they were missed in the project activities and were not participating in discussion about the solutions for their unique fertility issues [21]. The male engagement was limited in some cases such as various gender-related socio-cultural barriers [14] including not accepting females as a mentor, feeling shy while watching videos and being hesitant to participate and discuss reproductive health issues in any group activities because of stigma. Our results are similar to the findings from rural Kenya which has shown that socio-cultural barriers such as men’s reluctance not to change and having thought of RMNH as women’s business, led to the poor engagement of men [28]. The project captured the men aged 15–24 years from hard-to-reach communities as per the requirement of the project which targeted to reach AGYW, their husbands and families, and those who had poor access to RMNH services and information. Husbands of women who were recently married or who have recently had a baby were reached by the project. On the other hand, the project was not able to reach the male population who migrated to India and abroad as migrant laborers. Due to their engagement as daily wage workers, the men who were out of their homes and family members were not able to participate.

This study has a few limitations. The study gathered the perspective of women, family and stakeholders in Karnali Province. The context might be different in terms of geographical and rural urban variation within the country. However, the approaches and methods for male engagement can be applied in settings where men’s engagement is low. Because of the COVID-19 pandemic, we were not able to conduct face-to-face interviews and conduct focus groups discussions. The data collection was done entirely through a phone call with beneficiaries and key informants. This might have limited to capture the perception of the participants. However, we captured the information by taking adequate time in the phone call and following up with AGYW, their husband, and in-laws. In addition, social desirability bias as AGYW wanted to share that the positive sides of the project. To minimize such bias, we interviewed the participants explaining the objectives and put probing questions to validate their responses with beneficiaries and stakeholders.

## Conclusions

The use of interactive and participatory gender-transformative interventions within the project was found promising to improve engagement of husbands in RMNH, adopt gender equitable RMNH behavior that affect the health and well-being of AGYW. The socio-cultural barriers still existed in terms of engaging male in reproductive and maternal health, and reaching migrating men and those who work outside challenged the intervention to reach husbands. Future programs and approaches for engaging males in Nepal and other countries can draw based on the learnings from Healthy Transitions. Further research may be helpful to understand the dynamics of migration and reach out to migrating men in the context of Nepal.

## Data Availability

The datasets generated and analysed during this study will be available from the corresponding author on reasonable request.

## References

[1] Khatri RB, Karkee R (2018). Social determinants of health affecting utilisation of routine maternity services in Nepal: a narrative review of the evidence. Reprod Health Matters.

[2] Sekine K, Carter DJ (2019). The effect of child marriage on the utilization of maternal health care in Nepal: a cross-sectional analysis of demographic and health survey 2016. PLoS One.

[3] Ministry of Health, Nepal; New ERA; ICF. Nepal Demographic and Health Survey 2016. Kathmandu: Ministry of Health, Nepal; 2017. https://www.dhsprogram.com/pubs/pdf/fr336/fr336.pdf. Accessed 17 Aug 2021.

[4] Bello FO, Musoke P, Kwena Z (2019). The role of women’s empowerment and male engagement in pregnancy healthcare seeking behaviors in western Kenya. Women Health.

[5] Waiswa P, Kallander K, Peterson S, Tomson G, Pariyo GW (2010). Using the three delays model to understand why newborn babies die in eastern Uganda. Tropical Med Int Health.

[6] Glinski A, Schwenke C, O’Brien-Milne L, Farley K. Gender equality and male engagement: It only works when everyone plays | Align Platform. https://www.alignplatform.org/resources/gender-equality-and-male-engagement-it-only-works-when-everyone-plays. Accessed 9 Mar 2022.

[7] Fotso JC, Higgins-Steele A, Mohanty S (2015). Male engagement as a strategy to improve utilization and community-based delivery of maternal, newborn and child health services: evidence from an intervention in Odisha, India. BMC Health Serv Res.

[8] Mersha AG (2018). Male involvement in the maternal health care system: implication towards decreasing the high burden of maternal mortality. BMC Pregnancy Childbirth..

[9] Davis J, Vyankandondera J, Luchters S (2016). Male involvement in reproductive, maternal and child health: a qualitative study of policymaker and practitioner perspectives in the Pacific. Reprod Health.

[10] Ministry of Health and Population (2015). National Family Planning Costed Implementation Plan 2015–2020.

[11] Center for Reproductive Rights and Forum for Women, Law and Development (2018). Safe motherhood and reproductive health, rights act 2018.

[12] Family Welfare Division, Ministry of Health and Population. Nepal Safe Motherhood and Newborn Road Map 2030. https://fwd.gov.np/wp-content/uploads/2021/08/SMNH-Roadmap-2030-SGOP.pdf. Accessed 23 Oct 2021.

[13] Thapa DK, Niehof A (2013). Women's autonomy and husbands’ involvement in maternal health care in Nepal. Soc Sci Med.

[14] Sharma S, Kc B, Khatri A (2018). Factors influencing male participation in reproductive health: a qualitative study. J Multidiscip Healthc.

[15] Bhatta DN. Involvement of males in antenatal care, birth preparedness, exclusive breast feeding and immunizations for children in Kathmandu, Nepal. BMC Pregnancy Childbirth. 2013:13–4. 10.1186/1471-2393-13-14. 10.1186/1471-2393-13-14 PMC3558464 23324410

[16] Save the children. My first baby guide. Published 2013. https://www.savethechildren.org/content/dam/global/reports/health-and-nutrition/1st-baby-guide-brief.pdf. Accessed 19 Aug 2021.

[17] Implementing Pragati: Community games to increase fertility awareness and family planning use. Washington, D.C. Institute for Reproductive Health, Georgetown University; 2018. https://resource-centre-uploads.s3.amazonaws.com/uploads/Pragati+Manual+-+English_print.pdf. Accessed 17 Aug 2021.

[18] Institute of Reproductive Health, Georgetown University. FACT Project’s Roving auxiliary nurse midwife service delivery intervention. https://www.irh.org/roving-auxiliary-nurse-midwives/. Accessed 13 Nov 2021.

[19] Rampin R, Rampin V, DeMott S. Taguette (Version 1.0.0). Zenodo. 2021. 10.5281/zenodo.5111814.

[20] Thakuri DS, Thapa RK, Singh S, Khanal GN, Khatri RB (2021). A harmful religio-cultural practice (Chhaupadi) during menstruation among adolescent girls in Nepal: prevalence and policies for eradication. PLoS One.

[21] Mukherjee A, Lama M, Khakurel U, et al. Perception and practices of menstruation restrictions among urban adolescent girls and women in Nepal: a cross-sectional survey. Reprod Health. 2020:17–8. 10.1186/s12978-020-00935-6. 10.1186/s12978-020-00935-6 PMC7268527 32487096

[22] Joshi PR, Maharjan RK, Dawadi CK (2020). Nepalese women’s cultural beliefs and practices regarding postpartum period. J Sci Soc.

[23] Amatya P, Ghimire S, Callahan KE, Baral BK, Poudel KC (2018). Practice and lived experience of menstrual exiles (Chhaupadi) among adolescent girls in far-western Nepal. PLoS One.

[24] Mahon T, Tripathy A, Singh N. Putting the men into menstruation: the role of men and boys in community menstrual hygiene management. 2017. https://menstrualhygieneday.org/wp-content/uploads/2017/07/Putting-the-men-into-menstruation.pdf. Accessed Apr 21 2022.

[25] Mullany BC (2006). Barriers to and attitudes towards promoting husbands’ involvement in maternal health in Katmandu, Nepal. Soc Sci Med.

[26] Story WT (2012). Husbands’ involvement in delivery care utilization in rural Bangladesh: a qualitative study. BMC Pregnancy Childbirth.

[27] Conlin M, McKee S, Grenier S. Engaging males in maternal care “it took someone from the community to lead the change”. 2019. https://www.msh.org/sites/default/files/male-engagement-technicalbrief.pdf. Accessed 13 Aug 2021.

[28] Lusambili AM, Muriuki P, Wisofschi S (2021). Male involvement in reproductive and maternal and new child health: an evaluative qualitative study on facilitators and barriers from rural Kenya. Front Public Health.

